# Giving more detailed information about health insurance encourages consumers to choose compromise options

**DOI:** 10.3389/fpsyg.2023.1257031

**Published:** 2023-11-16

**Authors:** Stephen E. Chick, Scott A. Hawkins, David Soberman

**Affiliations:** ^1^INSEAD, Fontainebleau, France; ^2^Rotman School of Management, University of Toronto, Toronto, ON, Canada

**Keywords:** healthcare, insurance, compromise effect, unpacking, support theory, uncertainty

## Abstract

**Introduction:**

To investigate how the provision of additional information about the health events and procedures covered by a healthcare plan affect the level of coverage chosen by young adults taking their first full time job.

**Methods:**

University students were recruited for a study at two behavioral laboratories (one located at the University of Toronto and the other located at INSEAD-Sorbonne University in Paris) in which they imagine they are making choices about the healthcare coverage associated with the taking a new job in Chicago, Illinois. Every participant made choices in four categories: Physician Care, Clinical Care, Hospital Care, and Dental Care. Participants were randomly assigned to one of two conditions: Low Detail or High Detail coverage information and they chose between three levels of coverage: Basic, Enhanced, and Superior. The study took place in March 2017 with 120 students in Toronto and 121 students in Paris.

**Results:**

The provision of more detailed information about the health events and procedures covered by a healthcare plan leads to a compromise effect in which participants shift their choices significantly towards Enhanced (moderate coverage) from Basic (low coverage) and Superior (high coverage). The compromise effect was observed at both locations; however, Paris participants choose significantly higher levels of coverage than Toronto participants.

**Discussion:**

Providing more detail to employees about the health events and procedures covered by a healthcare plan will increase the fraction of employees who choose the intermediate level of coverage. It is beyond the scope of this study to conclude whether this is good or bad; however, in a context where employees gravitate to either insufficient or excessive coverage, providing additional detail may reduce these tendencies.

## 1. Introduction

In the US, many people receive healthcare insurance provided through employment or individual subscription. In countries like Canada and France, government-backed universal healthcare covers all residents.^1^ Nevertheless, government programs do not cover many elements of healthcare coverage. As a result, supplemental coverage offered by employers is important for employees as well as an important cost for employers. When selecting private healthcare insurance, enrollees typically make choices between different plans (often underwritten by different organizations) that provide different levels of coverage. The differences in plans include different deductibles or co-pays, different floors (when does the plan start to pay), and different ceilings (when does the plan’s coverage stop) across different healthcare procedures, drugs, and services. There are also differences in which services, drugs and procedures are covered (some plans cover the latest, most efficient procedures and medication; others are restricted to standard procedures and drugs that are less expensive). Plans vary substantially in the costs that the employee and the employer must pay.

The myriad of choices that must be made makes the selection of healthcare plans challenging for both employees and employers. This is especially true when people take their first full-time job. These choices are consequential – a recent study shows that the effect of health insurance on health status is significant, and it compounds over time (Barker and Li, 2020). We believe that a first step in improving healthcare coverage decisions is understanding the factors that drive these choices.

There is evidence that the amount of information provided to people affects their healthcare choices. The provision of Supplementary material to consumers about expected health costs has been found to induce customers to bear more risk, especially those in good health (Schoenbaum et al., 2001). Given our interest in assessing how people make healthcare choices when taking their *first job*, the quantity of information provided to employees might be used to manage populations that systematically over-insure or under-insure.

Our focus is to understand how providing more detailed information on the specific health events and procedures covered by a healthcare plan affects the level of coverage chosen by young adults who take a first, full-time job. Our study is nested in the context of healthcare management but uses concepts from behavioral economics and psychology to understand the choices that people make. Specifically, we examine the impact of providing added detail by unpacking macro-level information that can be inferred by individuals who think about the macro-category before making a choice. Unpacking a macro-category is not intended to provide additional information about health events covered by the plan; unpacking is designed to enumerate examples that are implicit in a category label. For example, coverage for mental healthcare can be unpacked by identifying the types of mental health issues included (e.g., social anxiety, eating disorders, depression, mood disorders, alcohol/drug addiction, and other mental healthcare issues). Participants do not need to reflect (or retrieve information from memory) to have details “top of mind” when they are provided explicitly.

Several constituencies will benefit from better understanding how healthcare choices are made. First, employees should be able to make better choices if they better understand the decisions they make (Barker and Li, 2020). Second, a better understanding of how employees make healthcare decisions will allow employers to improve the efficiency and performance of the menus they offer. Research has shown that healthcare is valuable to retain productive employees and to improve the health and productivity of the workforce (Whitmore et al., 2006). Third, healthcare plan providers and policy makers will benefit by better understanding how employees make healthcare plan choices at the start of a new job. In the following section, we provide a review of the literature related to our study.

## 2. Related research

Many factors are known to affect how people make healthcare coverage decisions. Barringer and Mitchell (1994) show that individual differences (e.g., income, gender, age, and marital status) can affect healthcare coverage choices. They also identify “risk minimization” in terms of future financial outlays (for uncovered treatments or in terms of high deductibles) as a key factor that affects choices. Additional work shows that people’s knowledge of their own health status affects the coverage they choose: people who have spent less on medical care prior to a coverage decision opt for less comprehensive plans while people who anticipate more medical spending opt for more comprehensive plans (Tchernis et al., 2006). These findings may be reinforced by a present bias that Wang and Sloan (2018) identify in the context of people making healthcare decisions. There are also studies which show that expertise (a person’s knowledge and understanding of issues related to healthcare) affects how information about healthcare is processed (Eber et al., 2021). This highlights the importance of controlling for demographic differences and differences in knowledge about healthcare and health status when assessing the impact of “detail” on choice.

As explained in the introduction, we want to understand how providing more detailed information on the specific health events and procedures covered by a healthcare plan affects the healthcare coverage chosen by first-time employees. We consider four different perspectives from psychology that can explain how added detail might affect decision-making in a context of uncertainty.

The first perspective regarding the effect of added detail is based on the observation that participants taking their first job have little experience in making decisions about both healthcare and healthcare coverage. Until the end of high school, most decisions related to healthcare and coverage are made by the students’ parents. Unless a student faces unusual health challenges after graduating from high school, this is a new decision. It is possible that added detail might be too much for students to process, leading to confused decision-making. Thus, the addition of detail might lead to increased randomness in the decisions of participants. This idea is based on how consumer confusion in healthcare affects decision-making (Lee and Lee, 2004; Gebele et al., 2014).

The second perspective regarding the effect of added detail is that it might alert participants to health events and procedures that are not otherwise recalled when assessing the risks associated with specific categories of healthcare coverage. Support theory argues that the judged probability of an event increases by unpacking that event (Tversky and Koehler, 1994). For example, describing the specific diagnostic tests covered by a healthcare plan increases the judged probability tests will be needed. Tversky and Kahneman (1973, 1974) demonstrated that people often use heuristics to assess probabilities, and these heuristics often lead to inconsistencies in judgements. Specifically, the provision of conjunctive details related to the probability of category membership can increase the perceived likelihood of category membership (Tversky and Kahneman, 1983). This tendency leads to a conjunctive fallacy that people exhibit when assessing the probability of joint events taking place. The quintessential example of this fallacy is Tversky and Kahneman’s “Linda, the bank teller” problem:

*Linda is 31 years old, single, outspoken and very bright. She majored in philosophy. As a student, she was deeply concerned with issues of discrimination and social justice. She is also participated in anti-nuclear demonstrations. Which is more probable?*

*1. Linda is a bank teller.*
*2. Linda is bank teller and is active in the feminist movement.*

More than 80% of participants choose option 2 even though the probability of two events occurring in conjunction is always less than or equal to the probability of one occurring alone, *P*(*A*∪*B*)≤min(P(*A*),P(*B*)), where A is “Linda is bank teller” and B is “Linda is active in the feminist movement. An extension to this idea is that the sum of probabilities related to mutually exclusive and exhaustive events should be the same independent of how the events are described (in the “Linda, the bank teller” example, the two events are not mutually exclusive). The standard context for a participant that chooses medical coverage for a variety of healthcare needs entails medical events that are generally mutually exclusive.

An important moderating factor is the degree to which people are familiar with the medical conditions covered by a healthcare plan. To be specific, Redden and Frederick (2011) show that unpacking can *reduce* the perceived likelihood of an event when people are familiar with the details. As noted in our discussion of the first perspective, unless the respondents are studying to enter medical school, it is unlikely that they are familiar with the conditions covered by a healthcare plan.

Support theory contributes to understanding judged probabilities by demonstrating situations where the probability judgements of respondents violate sub-additivity (Rottenstreich and Tversky, 1997; Idson et al., 2001). Support theory suggests that individuals might assign higher probabilities to unpacked healthcare events. In addition, there is evidence that unpacking leads to inconsistency in judgmentas it relates to length of an event (Liu et al., 2014). This might also affect the perceived need that participants have for healthcare coverage. In a consumption context, Tsai and Zhao (2011) show that the unpacking of a future consumption event can increase or decrease the time participants think they will spend on the event depending on whether the event is pleasant or not. Clearly, health problems are unpleasant but knowing that you are “covered” is pleasant. The indirect effects of unpacking are also important. For example, unpacking alternatives in conditions of uncertainty has been shown to improve a negotiator’s performance (Haselhuhn, 2015). It follows that understanding the effects of unpacking for choosing healthcare coverage, might allow an employer to improve the quality of choices made by new employees.

The third perspective that might explain the effect of added detail is to remind participants that several of the health events and procedures covered by the macro-categories do not apply to their situation (e.g., they are associated with older people). In a sense, the detail might have the effect of activating attributes of the coverage that are not valuable. Here, added detail about healthcare plans might induce consumers to bear more risk and choose less comprehensive coverage (Sloman et al., 2004).

The final perspective that can be used to explain the effect of added detail is that it might contribute to increased decision uncertainty for participants.^2^ For example, Simonson (1989), Simonson and Tversky (1992) demonstrate that people are more likely to choose an option when it is a compromise option (i.e., an alternative with attributes that are less extreme than other alternatives). The tendency of people to avoid extreme options has been demonstrated across a broad range of decision contexts (Neumann et al., 2016). Even in conditions where social norms influence the desirability of options, people tend to prefer compromise options (Du and Li, 2022).

Extensions to this research suggest that increased decision uncertainty (a more uniform distribution of probabilities across discrete choices) might lead to a compromise effect (Sheng et al., 2005). The precise mechanism that leads to high decision uncertainty is somewhat unclear, but one possibility is that the range of beliefs that participants have in terms of their needs for healthcare *ex ante* is wide. This implies that without detail, people who perceive their need for healthcare to be low would opt for minimal coverage and others who perceive their need for healthcare to be high (people who think of themselves as less healthy) would opt for maximal coverage. The addition of detail could make the first group think of procedures that they might need. For the second group, the addition of detail could remind participants of procedures that they would never need. These two effects will tend to increase uncertainty for the participants in which of the three levels of coverage is best. Following the literature on how decision uncertainty affects the choice of options from a menu, this might lead to a compromise.

For example, literature from economics and psychology highlights a distinction between higher risk in terms of expected outcomes (more variance in the expected outcomes) and more uncertainty in the assessment of probabilities associated with specific outcomes (Einhorn and Hogarth, 1985). When there is uncertainty associated with probability assessments, this is a situation of decision ambiguity. This distinction was first highlighted by Savage (1954) and later led to the Ellsberg paradox where participants exhibit preferences for gambles that, while identical in terms of expected utility, are different in terms of decision ambiguity (Ellsberg, 1961). It is possible that the addition of detail about health events and procedures covered by the healthcare plans might increase decision ambiguity (i.e., uncertainty about the probabilities of various healthcare coverage needs) for the participants. To the extent that more detail leads to less confidence in the information that people retrieve, a compromise effect might be more likely. This follows work by Chuang et al. (2012) which identifies situations where subjects with incomplete information are more likely to choose a compromise option than those with complete information.

Significant heterogeneity in how people react to decision ambiguity has been documented (Einhorn and Hogarth, 1985). Ambiguity aversion, although a stable phenomenon, has not been universally observed (Weber and Johnson, 2008).^3^ For example, in a relatively homogenous group of graduate students, Halevy (2007) finds three distinct segments (ambiguity-neutral, ambiguity-averse, and ambiguity-seeking) in terms of preferences over compound lotteries with different levels of ambiguity. Hence, heterogeneity in preferences for decision ambiguity also provides a plausible explanation for a compromise effect. As noted above, unpacking (i.e., providing more detail about each option in a choice set) may increase the ambiguity associated with the choices that subjects make.

In addition, Hogarth and Kunreuther (1989) find that participants exhibit ambiguity aversion for low probability of loss events and ambiguity preference for high probability of loss events. As a result, differences in the perceived starting point for participants may also provide an explanation for why participants who choose high levels of coverage and low levels of coverage, respectively might reduce or increase their preferred level of coverage in conditions of increased ambiguity. Given the potential of significant heterogeneity in the beliefs of people about the expected need for healthcare, Hogarth and Kunreuther (1989) provide another basis for predicting that added detail might lead to a compromise effect.

We next present the hypotheses that follow from the four perspectives about the possible effects of added detail (about the specific health events and procedures covered by the healthcare plan) on the level of healthcare coverage chosen by participants.

## 3. Hypothesis development

As explained in the introduction, the goal of this study is to measure the impact of providing more detail about the health events and procedures covered on the level of coverage that participants choose. The first possibility is that increased detail leads to confusion on the part of consumers, and this might lead to increased randomness in their decisions. We summarize this idea in Hypothesis 1.

*H1: Providing more detail about the conditions covered will lead to more random choices of level of healthcare coverage on the part of participants.*

When people believe there is an increased risk of expenses, which could occur when providing additional detail about the categories of coverage, they should choose higher levels of coverage (i.e., plans with higher monthly premiums and lower levels of out-of-pocket costs). We summarize this idea in Hypothesis 2.

*H2: Providing more detail about the conditions covered will lead participants to choose higher levels of insurance coverage.*

When people believe there is a decreased risk of expenses (because the added detail alerts people to a number of health events which do not seem relevant to their situation), they should choose lower levels of coverage. We summarize this idea in Hypothesis 3.

*H3: Providing more detail about the conditions covered will lead participants to choose lower levels of insurance coverage.*

Finally, when added detail contributes to increased decision uncertainty for participants, there is a significant body of research which predicts that people should be more likely to choose a compromise (less extreme) level of coverage. We summarize this in Hypothesis 4.

*H4: Providing more detail will lead participants to choose moderate levels of insurance coverage* (i.*e., a compromise effect).*

In summary, the objective of this study is to assess the impact of providing additional information about the health events and procedures covered by a healthcare plan on the choices that people make. The plan is to assess experimentally whether any of the four competing hypotheses are supported. In addition, a key objective of the study is to confirm the robustness of our findings across countries. The importance of validating general findings across cultures figures prominently in current research on healthcare (Tietschert et al., 2018). Our study will investigate whether the effect of providing more information to people who select healthcare is similar across two countries (Canada and France).

## 4. Methods

This study investigates the healthcare coverage choices that employees make when they begin working at a new job. University students are ideal for such an investigation because they are often close to the decision context we seek to investigate. The objective is to assess the relative willingness of new employees to self-insure as a function of the specificity of information that employees receive before making decisions.

The participants in this study were 241 undergraduate students from the University of Toronto and students in both 3- and 5-year programs at the INSEAD-Sorbonne University in Paris. There were 120 participants in Toronto and 121 participants in Paris, aged 18–30.

The range of ages is broad but 91% of the sample was less than 27 years of age. The broad range of age is driven by two factors. First, the age at which students start university in France is more variable because repeating years of school prior to university is 3 to 4 times more common in France than in Canada.^4^ Second, the vast majority of students in Paris were enrolled in a 5-year program which further increased the range of ages.^5^ As a result, the Paris sample had an average age that was 2.77 years higher (significant at *p* < 0.001) versus the Toronto sample (22.97 versus 20.20). Similarly, the standard deviation of the Paris sample was 1.3 years higher (significant at *p* < 0.001) than the Toronto sample (3.25 versus 1.99). Nevertheless, consistent with the recruiting guidelines, the vast majority of the sample had not worked full time.

Participants were paid to complete the 15-min study. The collection of data from two different groups allows us to assess the generality of our findings and to highlight important differences that may exist across cultures. Based on recent studies of similar effects (Beauchamp et al., 2020; Du and Li, 2022; Kim et al., 2022), the sample size provides sufficient power to assess the relevant hypotheses. The research reported here was approved by the University of Toronto and INSEAD-Sorbonne University Research Ethics Boards.

### 4.1. Materials

Participants were asked to imagine that they had just accepted a new job in the Chicago area, which requires them to make some individual choices about healthcare coverage. Chicago is in a foreign country for participants in both Toronto and Paris, and there is no national healthcare coverage in the US, so making insurance coverage decisions would by typical in such a move. They made decisions in four categories of healthcare coverage. The order of these decisions was randomized.

• Physician care (relates to the expenses incurred to see a family doctor)
• Clinical care (relates to expenses incurred in out-patient clinics)
• Hospital care (relates to expenses incurred in hospitals and for emergencies)
• Dental care (relates to expenses incurred due to services provided by dentists, orthodontists, periodontists, and dental surgeons)

For each type of healthcare coverage, participants chose among three levels of coverage: Basic, Enhanced and Superior. The test materials provide the following explanations for these levels.

(a) The **Basic Coverage** offers a lower monthly premium and covers a lower percentage of your healthcare costs (i.e., higher out-of-pocket costs that you must pay).
(b) The **Enhanced Coverage** offers a moderate monthly premium and covers a moderate percentage of your healthcare costs (i.e., moderate out-of-pocket costs that you must pay).
(c) The **Superior Coverage** offers a higher monthly premium and covers a higher percentage of your healthcare (i.e., lower out-of-pocket costs that you must pay).

The experimental materials explain that a participant’s total healthcare costs include monthly premiums (paid from the participant’s salary each month) and out-of-pocket costs (paid directly by the participant when healthcare costs are not fully covered by the chosen level of insurance coverage). For each healthcare choice, the participants saw a table that follows the format shown in Table 1. The choices are constructed such that expected total monthly costs of all three choices are identical. Thus, the modulation depends entirely on the participant’s desire to face uncertainty in expected monthly costs.

**TABLE 1:** The choice made by participants.

Plan description	Basic	Enhanced	Superior
Monthly premium deducted from salary	$20	$50	$80
Percentage of specified dental costs covered	60%	75%	90%
Expected monthly out-of-pocket expenses*	$100	$70	$40
Your choice (select one):	o	o	o

*Expected monthly out-of-pocket expenses are for the average plan member. Actual out-of-pocket expenses will depend on your specific healthcare needs.

After selecting the desired coverage levels for the four types of healthcare coverage and reporting the confidence in those choices, participants answered a set of demographic questions, beliefs about their relative health, subjective healthcare expertise, and long-term orientation.

For the measure of subjective expertise, four items were selected from previous research on measures of consumer knowledge (Mitchell and Dacin, 1996; Cowley and Mitchell, 2003). These items have been shown to be related to consumers’ ability to learn and organize information about various products/services. For the measure of Long-Term Orientation, six items were chosen from the Long-Term Orientation Scale (Beardon et al., 2006), which is related to the model of cross-cultural variation developed by Hofstede et al. (2010). These items measure the extent to which each participant is consistently more forward-looking or more present- or past-looking in their behaviors.

Finally, at the end of the study, participants answered a measure intended to assess their ability to understand and calculate expected monthly costs (including both monthly payroll deductions and expected monthly out-of-pocket expenses) for various insurance coverage plans.

### 4.2. Procedure

The study involved a 4 (type of insurance coverage) × 2 (level of detail: low vs. high) mixed design. The type of insurance coverage decision was manipulated within participants, while the level of detail was manipulated between participants. The level of detail was manipulated by unpacking one type of health event or procedure for each type of healthcare coverage (high detail) or simply providing the labels for the health events or procedures covered in each category (low detail). For example, medically necessary diagnostic tests covered in the physician care plan was unpacked to identify blood tests, electrocardiogram (EKG), ultrasound tests (sonography), X-rays, and CT scans, MRI scans (Magnetic Resonance imaging), and other medically necessary diagnostic tests. The specific health events and procedures that are covered in each type of coverage plan are provided in the Supplementary Appendix Part 1 (High Detail for the 4 types of coverage).

Participants took part in a lab session, and each was asked to complete an online questionnaire that was shown on a computer screen. The questionnaire for participants in the INSEAD lab were initially translated into French by a research assistant and reviewed by two of the principal investigators (SC and DS), who are fluent in both English and French, to ensure clarity and fidelity.

## 5. Results

As a first step to examine the results, we stacked the four types of healthcare coverage choices each participant makes (a total of 964 choices) and conducted a crosstab analysis to see whether there is a statistical difference between the decisions made under High and Low detail (Table 2).

**TABLE 2:** Detail × plan choice cross-tabulation.

			Choice			Total
			Basic	Enhanced	Superior	
Detail	High	Count	174	187	119	480
		% Within high	36.3%	39.0%	24.8%	100.0%
	Low	Count	206	141	137	484
		% Within low	42.6%	29.1%	28.3%	100.0%
Total		Count	380	328	256	964
		% For all choices	39.4%	34.0%	26.6%	100.0%

The Pearson Chi-Square for this cross-tabulation is 10.395, and with 2 degrees of freedom, this is statistically significant (*p* < 0.006). The table suggests that the High detail condition reduces the fraction of the participants that chose Basic and Superior and increases the fraction that chose Enhanced compared to the Low detail condition.

### 5.1. Evidence that high detail leads to increased randomness

We present several tests to assess whether there is evidence for increased levels of randomness (Hypothesis 1). The first is the Kolmogorov-Smirnov (KS) statistic for the distribution of choices made for both Low and High detail conditions. This statistic assesses whether the distribution is statistically different from what would be expected were people making choices randomly. The KS statistics for the Low detail and High detail, respectively are 0.275 and 0.236 (both statistics are significant with *p* < 0.001 and 484 and 480 degrees of freedom, respectively). However, the objective is to assess whether the randomness of decisions is increased by the High detail manipulation. To do this, we calculate two metrics of differences between discrete distributions.

The first is the symmetric Kullback–Leibler (KL) divergence, a statistical measure of how one probability distribution is different from a second, reference probability distribution, in terms of relative Shannon entropy (Kullback and Leibler, 1951). Colloquially, distributions that are close have a KL measure that is near zero. The symmetric KL divergence measure is:


$$KL_{Low\;Detail,\;Uniform}=\sum_{\text{i}=1}^{3}p_{i}^{Uniform}ln\left(\frac{p_{i}^{Uniform}}{p_{i}^{Low\;Detail}}\right)+\sum_{\text{i}=1}^{3}p_{i}^{Low\;Detail}ln\left(\frac{p_{i}^{Low\;Detail}}{p_{i}^{Uniform}}\right)$$

We compute *p*-values for this statistic using Monte Carlo methods (with 10,000 simulated KL measures under the null hypothesis for each test). The hypothesis that the Low Detail Distribution is uniformly distributed can be computed with simulations of random draws of populations using the sample size of the Low Detail data set.

Under the assumption that samples are uniform, we compute the distribution of empirical symmetric KL statistics, and then estimate the probability that the empirically sampled statistics are greater than the KL statistic computed with empirical probabilities from the Low detail data set. First, we compute the symmetric KL divergence from the Uniform to Low detail (0.0364), from the Uniform to High detail (0.0365) and from the Low detail to High detail (0.0433). The distribution of choices in Low and High detail is approximately the same distance from the Uniform distribution. Interestingly, the symmetric KL divergence between the High detail and Low detail distributions is greater than between either the Low detail or High detail distribution and the Uniform. While the High detail and Low detail distributions are significantly different from the Uniform distribution, these comparisons mean that the Low detail and High detail distributions differ from the Uniform distribution in ways that are distinct. Furthermore, the *p*-values for the symmetric KL statistics show that the Low Detail and High Detail distributions are significantly different from the uniform and from each other (*p* = 0.0003 for High detail, *p* = 0.0002 for Low Detail). The symmetric KL divergence analysis does not provide support for greater randomness in the choices of participants (Hypothesis 1).

We also test for similarities and differences of the distributions using the Hellinger distance (Hellinger, 1909). Like KL divergence, the Hellinger distance quantifies the difference between two probability distributions and is closer to 0 when two distributions are more similar:

H⁢e⁢l⁢lU⁢n⁢i⁢f⁢o⁢r⁢m⁢t⁢o⁢D⁢e⁢t⁢a⁢i⁢l=1-∑i=1NpiU⁢n⁢i⁢f⁢o⁢r⁢m⁢piD⁢e⁢t⁢a⁢i⁢l*

The Hellinger distances between High Detail and the Uniform distribution and between the Low Detail and Uniform distribution are 0.0675 and 0.675, respectively. Conversely, the Hellinger distance between the High Detail and Low Detail distributions is 0.0736. Like the symmetric KL divergence measure, the Hellinger distance shows that the distribution of choices for both conditions is approximately the same distance from the uniform distribution and that the distance between High detail and Low detail is greater than that between the Uniform and either of the Low detail and High detail distributions.

Using Monte Carlo simulations to estimate *p*-values for differences between each pair of distributions, we observe significance at levels observed for Hellinger distance (*p* = 0.0003 for each pairwise comparison). This is consistent with the findings based on KL divergence: the Low detail and High detail settings both lead to non-uniform selections, and the two detail levels lead to shifts away from randomness which are different.

In summary, three well-known measures to assess the similarity or difference between distributions (KS test, symmetric KL divergence, Hellinger distance) do not support the idea that added detail increases the tendency of the participants to choose randomly (Hypothesis 1).

### 5.2. Evidence that high detail leads to higher (lower) coverage

To assess whether High detail leads to higher (lower) coverage, we conducted a repeated-measures binomial logit analysis of the choices made by participants. Each participant is allocated to one of 4 conditions (Detail crossed by Location) and makes 4 choices (Physician, Clinical, Hospital and Dental). Because the choices of each participant in the four types of healthcare coverage may exhibit correlation, the repeated measures estimation is appropriate. We conduct two different estimations, which we call “from the bottom” (a tendency to choose Enhanced or Superior versus Basic) and “to the top” (a tendency to choose Superior versus Basic or Enhanced). We are interested in the effect that High Detail has on the choices that participants make. We summarize our expectations as a function of the two possibilities in Table 3.

**TABLE 3:** The expected effect of high detail.

	“From the bottom”	“To the top”
Higher coverage	Positive	Positive
Lower coverage	Negative	Negative

As noted earlier, Location was included as a control in the estimations. The type of choice (Physician, Clinical, Hospital and Dental), Gender, Age, Subjective Expertise, Relative Health, and Long-Term Orientation were also included as controls. Since Long-Term Orientation was not a significant factor in any of our statistical models, it will not be discussed further.

The repeated measures binary logit estimation for “from the bottom” and “to the top” are provided in the Supplementary Appendix Part 2. High detail is insignificant in both estimations (*p* = 0.073 and *p* = 0.403, respectively). In addition, there are significant effects of coverage type, subjective expertise and evidence that participants in France are more likely to choose Superior than are participants in Canada. In summary, we do not find support for either Hypotheses 2 or 3.

### 5.3. Evidence that high detail leads to a compromise effect

To assess whether High detail leads to a compromise effect, we conduct a repeated-measures, binomial logit estimation that assesses the tendency of participants to shift their choices “to the Middle” across conditions. The estimation shows that High detail has a significantly positive effect on the likelihood that Enhanced (a medium level of coverage) is chosen (*p* = 0.003). In addition, participants in Paris are significantly less likely to choose Enhanced (*p* = 0.035) and significantly more likely to choose Superior (*p* = 0.015) compared to participants in Toronto (See Supplementary Appendix Part 2 for details).

To further explore the evidence for a compromise effect, we conduct an analysis of the participants’ choices using a multinomial logit model in which each of the 4 healthcare choices are treated as independent decisions. The advantage of the multinomial logit model (over the binary logit model) is that each of the three choices are treated as independent outcomes predicted by the explanatory variables including Detail. As before, Location is included as a control in the estimations. The type of choice (Physician, Clinical, Hospital, and Dental), Gender, Age, Subjective Expertise and Relative Health are also included as controls. In Table 4, we present the results of the estimation with Enhanced as the reference category.

**TABLE 4:** Multinomial logit model to explain choice.

Choice^a^		B	Std. error	Wald	Df	Sig.
Basic	Intercept	1.415	0.876	2.610	1	0.106
	Age	0.018	0.029	0.361	1	0.548
	Gender	0.009	0.161	0.003	1	0.955
	Subjective expertise	−0.151	0.070	4.678	1	0.031
	Relative health	0.053	0.061	0.753	1	0.385
	[Location = I]	0.153	0.176	0.761	1	0.383
	[Location = R]	0^b^	.	.	0	.
	[Detail = H]	−0.458	0.157	8.496	1	0.004
	[Detail = L]	0^b^	.	.	0	.
	[Doctor dummy = 0.00]	−0.509	0.224	5.167	1	0.023
	[Doctor dummy = 1.00]	0^b^	.	.	0	.
	[Clinical dummy = 0.00]	−0.666	0.218	9.364	1	0.002
	[Clinical dummy = 1.00]	0^b^	.	.	0	.
	[Hospital dummy = 0.00]	−0.425	0.220	3.721	1	0.054
	[Hospital dummy = 1.00]	0^b^	.	.	0	.
Superior	Intercept	−0.727	0.946	0.592	1	0.442
	Age	−0.042	0.032	1.712	1	0.191
	Gender	−0.045	0.178	0.064	1	0.800
	Subjective expertise	0.119	0.076	2.438	1	0.118
	Relative health	0.106	0.066	2.616	1	0.106
	[Location = I]	0.615	0.194	10.061	1	0.002
	[Location = R]	0^b^	.	.	0	.
	[Detail = H]	−0.406	0.174	5.454	1	0.020
	[Detail = L]	0^b^	.	.	0	.
	[Doctor dummy = 0.00]	−0.115	0.229	0.251	1	0.616
	[Doctor dummy = 1.00]	0^b^	.	.	0	.
	[Clinical dummy = 0.00]	0.457	0.246	3.455	1	0.063
	[Clinical dummy = 1.00]	0^b^	.	.	0	.
	[Hospital dummy = 0.00]	0.190	0.232	0.671	1	0.413
	[Hospital dummy = 1.00]	0^b^	.	.	0	.

^a^The reference category is: enhanced. ^b^This parameter is set to zero because it is redundant.

In this analysis, High Detail significantly increases the choice of Enhanced compared to Basic (*p* = 0.004) and significantly increases Enhanced compared to Superior (*p* = 0.020). This is fully consistent with a Compromise Effect and reinforces the results of the repeated-measures binary logit analysis. We also find that the participants in the Paris location are more likely to choose Superior coverage compared to Enhanced (*p* = 0.002). This reinforces the finding from the binary logit estimations. The results also show that Subjective Expertise significantly reduces the likelihood that Basic coverage was chosen compared to Enhanced (*p* = 0.031). There are also significant effects associated with the type of choice participants were making, which underlines the importance of these controls. The remaining controls (Age, Gender, and Relative Health) are not significant predictors of choice.

In summary, both the repeated-measures, binomial logit estimation, and the multinomial logit analysis of the participants’ choices provide strong support for Hypothesis 4, which predicts a compromise effect when participants are exposed to High detail (i.e., an unpacked presentation of the insured health events and procedures for each category of healthcare). Next, we provide an analysis to elucidate this result.

### 5.4. Possible explanation for the compromise effect

In the study, we asked participants diagnostic questions related to psychographics, demographics, subjective expertise, relative health, and the estimated number of claims that they expect to make in the four categories for which they chose healthcare coverage. These questions were asked after the healthcare plan choice to replicate to the extent possible, the process that new employees go through when making healthcare choices. It is logical to expect that the estimated number of claims (across the 4 categories) has an impact on the level of coverage that people choose. As with the purchase of insurance, participants who expect a greater estimated number of claims are more likely to choose a higher level of coverage.

It is possible that the effect of providing more detail on the items in the healthcare plans causes a shift in the estimated total number of claims. To assess this possibility, we conducted a Means test on the full sample and on the participants in each country (as separate sub samples). Differences across conditions for the means are not significant (Supplementary Appendix Part 2).

To further assess the impact that the estimated total number of claims has on the level of coverage, we ran the estimations already conducted using the estimated number of claims as an additional predictor. The estimated total number of claims is a significant predictor of whether the participant chooses a higher level of coverage (Supplementary Appendix Part 2). However, the finding that High detail leads to a compromise effect (consistent with Hypothesis 4) remains significant when the estimated total number of claims is included.

The means comparison tests show that the standard deviation of the estimated total number of claims in the Low detail condition seems to be systematically smaller than in the High detail condition. To assess whether the difference is statistically significant, we calculate the Levene Statistic for the entire sample and for the Toronto and Paris sub-samples.

For the entire sample, the variance for Low detail is 33.017 and for High detail, it is 55.990. The value of the Levene Statistic is 6.137 (df = 239), which is significant (*p* = 0.014). For the Toronto sub-sample, the variance for Low detail is 29.495 and for High detail, it is 55.558. The value of the Levene Statistic is 4.536 (df = 118), which is significant (*p* = 0.035). For the Paris sub-sample, the variance for Low detail is 35.754 and for High detail, it is 55.370. The value of the Levene Statistic is 2.143 (df = 119) which is not significant (*p* = 0.146). However, the tendency of the manipulation to directionally increase the variance in the estimated total number of claims is consistent in both countries.

This analysis demonstrates that the High detail condition leads to higher variance in estimated total number of claims that the participants estimate for the coming year. Because participants are randomized across conditions in our study, we cannot assess the impact of High detail on an individual. However, higher variance across all choices made in the High Detail condition suggests less certainty about the number of claims a participant expects in this condition. This finding seems to be consistent with the High detail manipulation increasing the decision ambiguity associated with the healthcare choices being made. Specifically, the High detail condition seems to cause some participants to significantly reduce the estimated total number of claims and others to significantly increase the estimated total number of claims.

To further investigate whether the High Detail condition increases the variance (or decision ambiguity) in how participants predict future claims, we analyze the predicted number of claims for *specific items* within the four types of healthcare coverage (physician, clinical, hospital and dental). The specific items in the four types of coverage, respectively are estimated diagnostic test claims, estimated mental health claims, estimated physiotherapy/occupational therapy claims, and estimated dental claims. To isolate the effect of providing more detail in the High Detail conditions, the specific items were mentioned explicitly in both the Low and High Detail conditions.^6^

The analysis of the estimated number of sub-category claims is provided in the Supplementary Appendix Part 2. As with the analysis of total category claims, the difference in the means across conditions for the total sample is insignificant. However, the Levene Statistic is highly significant, implying that High detail leads to an increase in the variance of estimated sub-category claims. For the French and Canadian sub-samples, the variance of estimated sub-category claims also increases but the increase is insignificant. Because the expected number of sub-category claims is a less sensitive measure than total claims, this is perhaps unsurprising. In addition, the four sub-categories (where respondents estimate the expected number of claims) are *explicitly* mentioned in both the Low and High Detail conditions. Accordingly, the effects should be less pronounced. In any event, for the total sample, a similar increase in the variance of expected claims is observed.

We cannot determine the precise mechanism that leads to the compromise effect observed in the choices participants made under High Detail. It is possible that the explanation is based on people having significantly different reactions to higher decision ambiguity as noted by Halevy (2007). Alternatively, it may be that higher variance in the expected total number of claims leads participants to have a hypothetical optimal level of coverage that has a narrower spread. Some have argued this may lead to a compromise effect (Sheng et al., 2005).

### 5.5. Country effect on healthcare choices

The analyses presented provide preliminary evidence for a systematic difference in the choices that participants made as a function of their nationality. Most participants in the Toronto sample were Canadians (98/120) and most participants in the Paris sample were French (105/121) based on where the subjects attended high school. Our sample is almost perfectly balanced between Paris and Toronto and between High and Low Detail. Thus, a crosstab analysis is an appropriate statistical test for a difference between the choice proportions (Table 5).

**TABLE 5:** Location × choice cross tabulation.

			Choice			Total
			Basic	Enhanced	Superior	
Location	Paris	Count	188	148	148	484
		% Within location	38.8%	30.6%	30.6%	100.0%
	Toronto	Count	192	180	108	480
		% Within location	40.0%	37.5%	22.5%	100.0%
Total		Count	380	328	256	964
		% Within location	39.4%	34.0%	26.6%	100.0%

A lower proportion of Paris participants chose Basic and Enhanced coverage compared to Toronto participants. Conversely, a higher proportion of Paris participants chose Superior coverage (the highest level of coverage). The Chi-Square statistic for this Crosstab analysis is 9.398, which with 2 degrees of freedom is significant at *p* = 0.009. Thus, French participants were more likely to choose higher levels of coverage than Canadian participants.

This finding echoes findings in cross-cultural work regarding the appetite different nationalities have for uncertainty. Hofstede et al. (2010) identify Uncertainty Avoidance (the extent to which members of a culture feel threatened by uncertain or unknown situations) as a value dimension of cross-cultural variability. On this index (where lower scores mean lower Uncertainty Avoidance), Canada scores 48 and France scores 86 (the range extends from 8 to 112). By choosing higher levels of coverage, French participants reduce their exposure to uncertainty consistent with the measures of Hofstede et al. (2010).

We should qualify this finding by reminding readers of the significant difference in the age distribution of the Paris and Toronto samples. As discussed in the section “4. Methods,” the Paris sample was both older and broader than the Toronto sample. Age is a possible explanatory factor for the difference across countries; however, age was insignificant in every model analyzed to explain the choices made by the subjects. We suspect that a study which has greater breadth in the age variable may find differences based on how life-experience (illness and/or employment) affects health insurance choices.

## 6. Discussion

Our goal is to investigate how the provision of additional information about health events and procedures covered by a healthcare plan affect the level of coverage chosen by young adults who take their first full time job. Better understanding of this process will help employees make “good” healthcare choices and guide employers in the design of the menus they offer to employees. In addition, healthcare plan providers and policy makers will benefit from better understanding of the drivers of choice from the perspective of employees.

Conventional wisdom suggests that, with better information, decision makers will make better decisions. However, when it comes to employees making decisions about the level of healthcare to choose, a critical feature of the context is that first-time employees are not well informed about healthcare (in general) or insurance.

The study shows that the provision of more detailed information about the health events and procedures covered by a healthcare plan leads to a compromise effect in which participants shift their choices significantly toward Enhanced coverage (the middle choice) from Basic coverage (the low choice) and Superior coverage (the high choice). This tendency exists even when controlling for other possible influencing factors such as the nature of the decision (physician, clinical, hospital or dental), location (Toronto versus Paris), and various demographic and individual difference measures. Given that some people have a tendency to under-insure and others have a tendency to over-insure, this finding can be used to increase the likelihood that employees make better choices in terms of healthcare coverage. This echoes the findings of Sharpe et al. (2008) regarding the use of extremeness aversion to combat obesity.

We also find that High detail leads to an increase in the variance of the estimated number of claims across the four categories for which each participant chooses coverage. This suggests that High detail leads to higher decision ambiguity associated with the healthcare choices being made. Specifically, the manipulation causes some participants to significantly reduce the estimated number of claims and others to significantly increase it. While our study cannot conclude that increased variance in the estimated number of claims is the cause of the compromise effect, there are several theoretical explanations for the compromise effect that are consistent with this dynamic.

In addition, our analyses show that French participants were more likely to choose higher levels of coverage than Canadian participants. This follows findings in cross-cultural work regarding the appetite different nationalities have for uncertainty. This finding points to the need to account for culture when firms design healthcare options for new employees. This is true for companies that operate in countries where there are significant differences in Uncertainty Avoidance according to the work of Hofstede et al. (2010).

### 6.1. Limitations

Our study does have limitations. First, the effects demonstrated in our experiments use hypothetical choices of healthcare coverage by participants. It is possible for there to be a difference between the hypothetical choices and actual decision-making by new employees. However, we control for this by identifying participants who are likely to be making similar choices soon. In addition, the validity of the hypothetical choices being representative relies on participants not having an incentive to distort or answer differently that they would were they making the actual decision. We cannot think of a reason for why participants would want to distort their answers.

Second, our research is focused on students who are in the later stages of their undergraduate studies. Because people change jobs significantly more today than in the past, it may be risky to generalize our findings to older people who have more experience with choices like the ones examined in this study.

Finally, participants in this study are enrolled in university. There is substantial research which demonstrates significant differences across populations based on their level of education. Accordingly, it may be risky to generalize our findings to people who are taking their first job right after high school or after (shorter) technical apprenticeships. However, recent reports by the OECD indicate that significantly more than 50% of Canadian adults have some level of college education and in France, the percentage is 34%. In other words, even if the relevance of the findings is restricted to those who are college educated, they remain important because a large fraction of the population in OECD countries continues their education after high school.^7^

## Data availability statement

The raw data supporting the conclusions of this article will be made available by the authors, without undue reservation.

## Ethics statement

The studies involving humans were approved by the INSEAD-Sorbonne University Research Ethics Board and the University of Toronto Social Sciences, Humanities and Education Research Ethics Board. The studies were conducted in accordance with the local legislation and institutional requirements. The participants provided their written informed consent to participate in this study.

## Author contributions

SC: Conceptualization, Formal analysis, Funding acquisition, Investigation, Methodology, Project administration, Supervision, Validation, Writing – original draft, Writing – review and editing. SH: Conceptualization, Formal analysis, Investigation, Methodology, Project administration, Supervision, Validation, Writing – original draft, Writing – review and editing. DS: Conceptualization, Formal analysis, Funding acquisition, Investigation, Methodology, Project administration, Supervision, Validation, Writing – original draft, Writing – review and editing.

## Footnotes

1 Background on the healthcare systems of various countries can found in Schneider et al. (2017).

2 Recent research shows that when people make risky decisions (like buying health insurance), the dispersion and skewness of expected utility affects decisions (Bayrak and Hey, 2020).

3 The link between the tendency of people to compromise and their appetite for risk is topic of considerable interest. In fact, the strength of the compromise effect can be used to estimate the risk preferences of individual participants (Beauchamp et al., 2020).

4 Pisa statistics on the prevalence of grade repetition across countries https://www.oecd.org/pisa/keyfindings/Vol4Ch2.pdf.

5 French government statistics on the fraction of university students that complete 5 versus 3-year programs. https://publication.enseignementsup-recherche.gouv.fr/eesr/8EN/EESR8EN_ES_17-student_trajectories_and_pass_rates_at_universities.php.

6 Originally, these questions were intended to check that participants were reading the plan descriptions carefully; however, given the findings with respect to total claims, these questions provide the opportunity to conduct a robustness check on the finding that High detail led to an increase in the variance of the estimated total number of claims but not the mean.

7 These percentages are found at https://gpseducation.oecd.org/CountryProfile?primaryCountry~=~CAN&treshold~=~10&topic~=~EO and https://www.oecd.org/education/skills-beyond-school/EAG2016-France-Eng.pdf.
