# Supplementary material for: Giving more detailed information about health insurance encourages consumers to choose compromise options
Source: Front Psychol. 2023 Nov 16;14:1257031. doi: 10.3389/fpsyg.2023.1257031 (PMC10693418; doi:10.3389/fpsyg.2023.1257031)
Supplement: Supplementary file 1 [file Data_Sheet_1.PDF]

Online Appendix Part 1

High Detail Stimuli

Your Healthcare Coverage Options

In order to meet the varying needs of our employees, we offer three different levels of coverage for each type of health insurance: Basic, Enhanced, and Superior Coverage.

> The **Basic Coverage** offers a lower monthly premium and covers a lower percentage of your healthcare costs (i.e., higher out-of-pocket costs that you must pay).
>
> The **Enhanced Coverage** offers a moderate monthly premium and covers a moderate percentage of your healthcare costs (i.e., moderate out-of-pocket costs that you must pay).
>
> The **Superior Coverage** offers a higher monthly premium and covers a higher percentage of your healthcare (i.e., lower out-of-pocket costs that you must pay).

Your **total healthcare costs** will include your *monthly premiums*, which will be paid from your salary each month, and your *out-of-pocket costs*, which you will pay whenever you incur healthcare costs that are not completely covered by your chosen level of insurance coverage. Your total annual healthcare costs will depend on the plans that you choose and your specific healthcare needs.

We encourage you to carefully review the explanations of expenses covered under the various plans so that you can make the best choices for your personal needs.

Physician Care

Your Physician Care insurance will cover a percentage of the expenses associated with routine medical treatment by a primary-care (family) physician.

Regular Care from your Physician

The **coverage** in this plan will pay the following specific physician expenses:

- ✓ annual physical evaluation
- ✓ physician visits for any condition that affects normal functioning
- ✓ vaccinations
- ✓ medically-necessary diagnostic tests
  - ➢ blood tests
  - ➢ electrocardiogram (EKG)
  - ➢ Ultrasound tests (Sonography)
  - ➢ X-Rays and CT scans
  - ➢ MRI scans (Magnetic Resonance Imaging)
  - ➢ other medically-necessary diagnostic tests

Please think carefully about your personal needs for the types of **regular care from your physician** listed above and **select your preferred Physician Care plan**.

**Plan Description**	**Basic**	**Enhanced**	**Superior**
Monthly Premium Deducted from Salary	$80	$105	$130
Percentage of Specified Dental Costs Covered	60%	70%	80%
Expected Monthly Out-of-pocket Expenses*	$65	$40	$15
**Your Choice (select one):**	o	o	o

*Expected monthly out-of-pocket expenses are for the average plan member. Actual out-of-pocket expenses will depend on your specific healthcare needs.

Clinical Care

Your Clinical Care insurance will cover a percentage of the expenses associated with medical treatments provided in a clinical setting (i.e., as an out-patient).

Treatment in a Clinical Setting

The **coverage** in this plan will pay the following specific clinical expenses:

- ✓ physician services
- ✓ nursing care
- ✓ out-patient rehabilitation facilities
- ✓ social services
- ✓ mental healthcare
    - ➢ social anxiety
    - ➢ eating disorders
    - ➢ depression
    - ➢ mood disorders
    - ➢ alcohol/drug addiction
    - ➢ other mental healthcare issues

Please think carefully about your personal needs for the types of **treatment in a clinical setting** listed above and **select your preferred Clinical Care plan**.

**Plan Description**	**Basic**	**Enhanced**	**Superior**
Monthly Premium Deducted from Salary	$70	$80	$90
Percentage of Specified Dental Costs Covered	50%	60%	70%
Expected Monthly Out-of-pocket Expenses*	$65	$55	$45
**Your Choice (select one):**	o	o	o

*Expected monthly out-of-pocket expenses are for the average plan member. Actual out-of-pocket expenses will depend on your specific healthcare needs.

Hospital Care

Your Hospital Care insurance will cover a percentage of the expenses associated with medical treatments provided in a hospital setting (i.e., as an in-patient).

Treatment in a Hospital Setting

The **coverage** in this plan will pay the following specific hospital expenses:

- ✓ physician services
- ✓ nursing care
- ✓ semi-private facilities
- ✓ inpatient medications and treatments
- ✓ physical or occupational therapy
  - ➢ injury screening
  - ➢ back, shoulder or neck pain
  - ➢ leg or foot pain
  - ➢ joint pain
  - ➢ pre- and post-operative care
  - ➢ other situations requiring physical or occupational therapies

Please think carefully about your personal needs for the types of **treatment in a hospital setting** listed above and **select your preferred Hospital Care plan**.

Plan Description	Basic	Enhanced	Superior
Monthly Premium Deducted from Salary	$120	$160	$200
Percentage of Specified Dental Costs Covered	75%	85%	95%
Expected Monthly Out-of-pocket Expenses*	$115	$75	$35
**Your Choice (select one):**	o	o	o

*Expected monthly out-of-pocket expenses are for the average plan member. Actual out-of-pocket expenses will depend on your specific healthcare needs.

Dental Care

Your Dental Care insurance will cover a percentage of the expenses associated with treatment by a dentist or dental practitioner.

Regular Care from your Dentist

The **coverage** in this plan will pay the following specific dental expenses:

- ✓ routine and emergency diagnostic tests or procedures
- ✓ preventative care including semi-annual cleaning, scaling, or polishing
- ✓ surgical procedures including root canals, crowns, or implants
- ✓ restorative care to repair tooth damage or decay
  - ➢ dental fillings and repairs
  - ➢ chipped and broken tooth repair
  - ➢ tooth/root extraction
  - ➢ pain control
  - ➢ teeth whitening
  - ➢ other restorative care to repair tooth damage or decay

Please think carefully about your personal needs for the types of **regular care from your dentist** listed above and **select your preferred Dental Care plan**.

Plan Description	Basic	Enhanced	Superior
Monthly Premium Deducted from Salary	$20	$50	$80
Percentage of Specified Dental Costs Covered	60%	75%	90%
Expected Monthly Out-of-pocket Expenses*	$100	$70	$40
**Your Choice (select one):**	o	o	o

*Expected monthly out-of-pocket expenses are for the average plan member. Actual out-of-pocket expenses will depend on your specific healthcare needs.

Online Appendix Part 2

Binomial Logit Estimations for “from the bottom”, “to the top” and “to the middle”

First, we provide the repeated measures binary logit estimation for “from the bottom”.

Parameter	B	Std. Error	Hypothesis Test		
			Wald Chi-Square	df	Sig.
(Intercept)	-.799	.8749	.835	1	.361
[Location_num=.00]	-.111	.1799	.377	1	.539
[Location_num=1.00]	0[a]	.	.	.	.
[Detail=H]	-.288	.1606	3.221	1	.073
[Detail=L]	0[a]	.	.	.	.
Age	.035	.0319	1.235	1	.267
Gender	.029	.1624	.033	1	.856
Relative Health	.006	.0670	.009	1	.924
Subjective Expertise	-.202	.0763	7.021	1	.008
doctor dummy	.455	.1806	6.338	1	.012
clinical dummy	.852	.1883	20.490	1	.000
hospital dummy	.508	.1860	7.451	1	.006
(Scale)	1				

The effect of High detail has a slight negative effect on the likelihood that Basic is chosen but the effect is only marginally significant (7.3% level). There are significant effects of coverage type. Subjective expertise is also observed to have significant negative effect on the likelihood that Basic is chosen.

Second, we provide the repeated measures binary logit estimation for “to the Top”.

Parameter	B	Std. Error	Hypothesis Test		
			Wald Chi-Square	df	Sig.
(Intercept)	-.827	.9130	.820	1	.365
[Location_num=.00]	.535	.2144	6.220	1	.013
[Location_num=1.00]	0[a]	.	.	.	.
[Detail=H]	-.164	.1965	.699	1	.403
[Detail=L]	0[a]	.	.	.	.
Age	-.051	.0352	2.106	1	.147
Gender	-.046	.2040	.051	1	.821
Relative Health	.078	.0729	1.147	1	.284
Subjective Expertise	.199	.0866	5.275	1	.022
doctor dummy	-.139	.1765	.621	1	.430
clinical dummy	-.801	.2063	15.090	1	.000
hospital dummy	-.397	.1752	5.134	1	.023
(Scale)	1				

The effect of High detail has a negative effect on the likelihood that Superior is chosen but the effect is not significant. In addition, participants in Paris are significantly more likely to choose Superior than are participants in Toronto (1.3% significance level). There are significant effects of coverage type and subjective expertise is observed to positively affect the likelihood that Superior is chosen.

Third, we provide the repeated measures binary logit estimation for “to the Middle”.

Parameter	B	Std. Error	Hypothesis Test		
			Wald Chi-Square	df	Sig.
(Intercept)	-.531	.7144	.552	1	.457
[Location_num=.00]	-.343	.1624	4.458	1	.035
[Location_num=1.00]	0[a]	.	.	.	.
[Detail=H]	.434	.1475	8.658	1	.003
[Detail=L]	0[a]	.	.	.	.
Age	.007	.0276	.061	1	.804
Gender	.014	.1508	.009	1	.924
Relative Health	-.075	.0559	1.824	1	.177
Subjective Expertise	.040	.0669	.365	1	.546
doctor dummy	-.321	.1875	2.929	1	.087
clinical dummy	-.223	.1969	1.284	1	.257
hospital dummy	-.147	.1929	.582	1	.446
(Scale)	1				

The effect of High detail has a significantly positive effect on the likelihood that Enhanced is chosen and the effect is significant at the 0.3% level. The only other significant effect is that participants in Paris are significantly less likely to choose Enhanced than participants in Toronto.

Analysis of the Mean Number of Claims

Total Sample

	BinDetail	N	Mean	Std. Deviation	Std. Error Mean
total_claims	if H	120	12.0417	7.48264	.68307
	if L	121	11.3140	5.74606	.52237

Is the difference significant (Equal variances not assumed)?

T	df	Sig. (2-tailed)	Mean Difference
.846	223.190	.398	.72762

Toronto Sample

	BinDetail	N	Mean	Std. Deviation	Std. Error Mean
total_claims	if H	60	13.0333	7.45374	.96227
	if L	60	12.1167	5.43090	.70113

Is the difference significant (Equal variances not assumed)?

T	df	Sig. (2-tailed)	Mean Difference
.770	107.870	.443	.91667

Paris Sample

	BinDetail	N	Mean	Std. Deviation	Std. Error Mean
total_claims	if H	60	11.0500	7.44112	.96065
	if L	61	10.5246	5.97943	.76559

Is the difference significant (Equal variances not assumed)?

T	df	Sig. (2-tailed)	Mean Difference
.428	112.944	.670	.52541

Estimations with the Number of Claims as a Predictor

We show below the results of the multinomial logit estimation with Enhanced as the reference category.

choice[a]		B	Std. Error	Wald	df	Sig.
Basic	Intercept	.582	.778	.561	1	.454
	number of claims	-.127	.038	11.286	1	.001
	Age	.017	.029	.337	1	.562
	Gender	.007	.160	.002	1	.967
	Relative Health	.036	.061	.348	1	.555
	Subjective Expertise	-.127	.071	3.250	1	.071
	[Detail=H]	-.438	.157	7.802	1	.005
	[Detail=L]	0[b]	.	.	0	.
	[Location_num=.00]	.070	.178	.156	1	.693
	[Location_num=1.00]	0[b]	.	.	0	.
Superior	Intercept	-1.128	.877	1.656	1	.198
	number of claims	.202	.036	31.882	1	.000
	Age	-.034	.033	1.051	1	.305
	Gender	-.007	.183	.001	1	.971
	Relative Health	.133	.068	3.858	1	.050
	Subjective Expertise	.056	.078	.514	1	.473
	[Detail=H]	-.447	.178	6.287	1	.012
	[Detail=L]	0[b]	.	.	0	.
	[Location_num=.00]	.695	.199	12.160	1	.000
	[Location_num=1.00]	0[b]	.	.	0	.

a. The reference category is: Enhanced.
b. This parameter is set to zero because it is redundant.

As with the multinomial model without the estimated number of claims as a predictor, a strong compromise effect of the Detail manipulation is evident. In addition, the tendency of Paris participants to choose Superior coverage compared to Enhanced is highly significant (p=.000). The effect of the estimated number of claims is highly significant (claims is negatively associated with choosing Basic and positively associated with choosing Superior).

Nevertheless, there is no evidence that the Low/High Detail manipulation affects the choices of participants by increasing the estimated number of claims. As noted above, the differences across the sample are insignificant.

Analysis of the Mean and Variance of the Number of Sub-Category Claims

To further assess whether the High Detail condition increases the mean and/or variance (or decision ambiguity) in how participants predict future claims, we analyze the predicted number of claims for *specific items* within the four types of healthcare coverage (physician, clinical, hospital and dental). The specific items in the four types of coverage respectively are estimated diagnostic test claims, estimated mental health claims, estimated physiotherapy/occupational therapy claims, and estimated restorative tooth claims. To isolate the effect of providing more detail in the High Detail conditions, the specific items were mentioned explicitly in both the Low and High Detail conditions. Originally, the purpose of these questions was to check that participants were reading the plan descriptions carefully; however, given our findings with

respect to total claims, these questions provide the opportunity to conduct a robustness check on the finding that High detail led to an increase in the variance of the estimated total number of claims (but not its mean).

The analysis of the estimated number of sub-category claims is provided below. As with the analysis of total category claims, the difference in the means across conditions for the total sample is insignificant.

The specific items in the four macro categories respectively were estimated diagnostic test claims, estimated mental health claims, estimated physiotherapy/occupational therapy claims and restorative tooth claims.

Total Sample

	BinDetail	N	Mean	Std. Deviation	Std. Error Mean
totalsubcategory_claims	if H	120	11.6333	6.74996	.61618
	if L	121	10.3471	5.87326	.53393

Is the difference significant (Equal variances not assumed)?

t	Df	Sig. (2-tailed)	Mean Difference
1.578	233.979	.116	1.28623

Toronto Sample

	BinDetail	N	Mean	Std. Deviation	Std. Error Mean
totalsubcategory_claims	if H	60	12.1667	6.54105	.84445
	if L	60	11.0167	5.73568	.74047

Is the difference significant (Equal variances not assumed)?

t	Df	Sig. (2-tailed)	Mean Difference
1.024	116.020	.308	1.15000

Paris Sample

	BinDetail	N	Mean	Std. Deviation	Std. Error Mean
totalsubcategory_claims	if H	60	11.1000	6.96651	.89937
	if L	61	9.6885	5.97924	.76556

Is the difference significant (Equal variances not assumed)?

t	df	Sig. (2-tailed)	Mean Difference
1.195	115.729	.235	1.41148

The above analysis demonstrates that the unpackaging of detailed information (the High Detail condition) does not have a significant effect on increasing the number of sub-category claims. This echoes the findings with regards to total category claims.

Analysis of the Homogeneity of Variance for the Number of Sub-Category Claims

It is evident from the means comparison tests that the standard deviation of the estimated number of claims in the Low Detail condition seems to be systematically smaller than the standard deviation of the estimated number of claims in the High Detail condition. To assess whether the difference is statistically significant, we calculate the Levene Statistic for the total sample and for the Toronto and Paris subsamples.

Total Sample

Variance for Low Detail is 34.495 and for High Detail is 45.562. We then calculate the Levene Statistic to compare the Variance and find that the difference is highly significant (p=.049)

		Levene Statistic	df1	df2	Sig.
totalsubcategory_claims	Based on Mean	3.929	1	239	.049

Toronto Sample

Variance for Low Detail is 32.898 and for High Detail is 42.7858. We then calculate the Levene Statistic to compare the Variance and find that the difference is insignificant (p=.168).

		Levene Statistic	df1	df2	Sig.
totalsubcategory_claims	Based on Mean	1.921	1	118	.168

Paris Sample

Variance for Low Detail is 35.751 and for High Detail is 48.532. We then calculate the Levene Statistic to compare the Variance and find that the difference in the Paris sample is not significant (p=.136). However, the tendency of the manipulation to increase the variance in the estimated number of claims is consistent across both countries.

		Levene Statistic	df1	df2	Sig.
totalsubcategory_claims	Based on Mean	2.258	1	119	.136

For the French and Canadian sub-samples, the difference in the variance in sub-category claims within each of the two sub-samples is not significant yet directionally the increase in the variance

is consistent with a contextual increase in Decision uncertainty as was the case for total category claims. Because the expected number of sub-category claims is a less sensitive measure than total claims, this is perhaps unsurprising. Moreover, the four sub-category claims included in this test are *explicitly* mentioned in both the Low and High Detail conditions. Accordingly, the effects should be less pronounced. In any event, for the total sample, the Levene Statistic is significant.
